# Phenylethanoid Glycosides From Callicarpa kwangtungensis Chun Attenuate TNF-α-Induced Cell Damage by Inhibiting NF-κB Pathway and Enhancing Nrf2 Pathway in A549 Cells

**DOI:** 10.3389/fphar.2021.693983

**Published:** 2021-07-07

**Authors:** Jing-Na Zheng, Jian-Yi Zhuo, Juan Nie, Yan-Lu Liu, Bao-Yi Chen, Ai-Zhi Wu, Yu-Cui Li

**Affiliations:** ^1^ School of Pharmaceutical Sciences, Guangzhou University of Chinese Medicine, Guangzhou, China; ^2^ Department of Pharmacy, The First Affiliated Hospital/School of Clinical Medicine of Guangdong Pharmaceutical University, Guangzhou, China

**Keywords:** acute lung injury, callicarpa kwangtungensis chun, TNF-α, NF-κB, Nrf2

## Abstract

**Background:** Acute lung injury (ALI) is a complicated and severe lung disease, which is often characterized by acute inflammation. Poliumoside (POL), acteoside (ACT) and forsythiaside B (FTB) are phenylethanoid glycosides (PGs) with strong antioxidant, anti-inflammatory, and anti-apoptotic properties, which are extracted from *Callicarpa kwangtungensis Chun* (CK). The aim of this study was to investigate the protective effects of POL, ACT, and FTB against TNF-α-induced damage using an ALI cell model and explore their potential mechanisms.

**Methods and Results:** MTT method was used to measure cell viability. Flow cytometry was used for detecting the apoptosis rate. Reactive oxygen species (ROS) activity was determined using fluorescence microscope. The expression of mRNA in apoptosis-related genes (Caspase 3, Caspase 8, and Caspase 9) were tested by qPCR. The effects of POL, ACT, FTB on the activities of nuclear factor erythroid-2 related factor 2 (Nrf2), nuclear factor kappa-B (NF-κB) and the expression of their downstream genes were assessed by western blotting and RT-PCR in A549 cells. In the current study, POL, ACT, and FTB dose-dependently attenuated TNF-α-induced IL-1β, IL-6 and IL-8 production, cell apoptosis, the expression of apoptosis-related genes (Caspase 3, Caspase 8, and Caspase 9) and ROS activity. POL, ACT, and FTB not only increased in the mRNA levels of antioxidative enzymes NADPH quinone oxidoreductase (NQO1), glutamate cysteine ligase catalytic subunit (GCLC), heme oxygenase (HO-1), but also decreased the mRNA levels of IL-1β, IL-6 and IL-8. Furthermore, they upregulated the expression of Keap1 and enhanced the activation of Nrf2, while decreased the expression of phosphor-IκBα (*p*-IκBα) and nuclear p65. In addition, no significant changes were observed in anti-inflammatory and antioxidant effects of POL, ACT, FTB following Nrf2 and NF-κB p65 knockdown.

**Conclusion:** Our study revealed that POL, ACT, and FTB alleviated oxidative damage and lung inflammation of TNF-α-induced ALI cell model through regulating the Nrf2 and NF-κB pathways.

## Introduction

Acute lung injury (ALI), one of the common critical diseases in the clinical respiratory department, is often characterized by acute inflammation with complex etiology and mechanism (Butt et al., 2016). The common clinical manifestations include dyspnea, acute pulmonary edema, progressive pulmonary fibrosis and hypoxemia. In severe cases, it frequently progresses to acute respiratory distress syndrome (ARDS) or even death (Komiya et al., 2017). The mortality may be up to 25–40%. At present, it is believed that the key mechanism of ALI involves the imbalance of inflammatory response, dysregulation of oxidative stress, uncontrolled activation of the coagulation system, disorder of immune responses, and apoptosis in the lung (Johnson and Matthay, 2010). However, its pathogenesis has not yet been clearly understood in modern medicine. Moreover, no effective treatment method and satisfactory therapeutic measure exists for patients as far (Chen et al., 2015). Some agents of natural origin, such as medicinal plants and their secondary metabolites, have been reported to be potential alternatives for ALI treatment because of many pharmacological activities and few side effects (Favarin et al., 2013).

Combined with the current findings in the pathogenesis of ALI, it was found that ALI was alleviated by blocking the network of related signaling pathways in the body specifically and regulating various cytokines (Lelli et al., 2017). Particularly, the NF-κB and Keap1-Nrf2 pathways have been commonly used to investigate the mechanisms of ALI (Guo and Ward, 2007; Chopra et al., 2009; Lyu et al., 2012). In fact, there is growing evidence that Keap1-Nrf2 pathway and NF-κB pathway work interdependently, not independently, and there is a close interaction between them, forming a “crosstalk” (Yerra et al., 2013; Wardyn et al., 2015). Earlier studies have indicated that NF-κB of cytoplasm always binds to the inhibitory protein IκBs at rest during normal life. Once the phosphorylation and degradation of IκB are stimulated by a variety of factors, such as cytokines (TNF-α, IL-1β), is triggered and NF-κB subunits will be transferred into the cell nucleus, which leads to activation of a multitude of genes, such as apoptosis inhibitors and pro-inflammatory genes (Wang et al., 2017). Under normal physiological conditions, kelch-like ECH-related protein-1 (Keap1) and Nrf2 are in a state of binding dynamic equilibrium. In addition, oxidative stress could disrupt the binding of Nrf2/Keap1 complex, and rapidly translocate Nrf2 from the cytoplasm into the nucleus, activating a series of antioxidant genes (Jiang et al., 2016).

**FIGURE 1:**
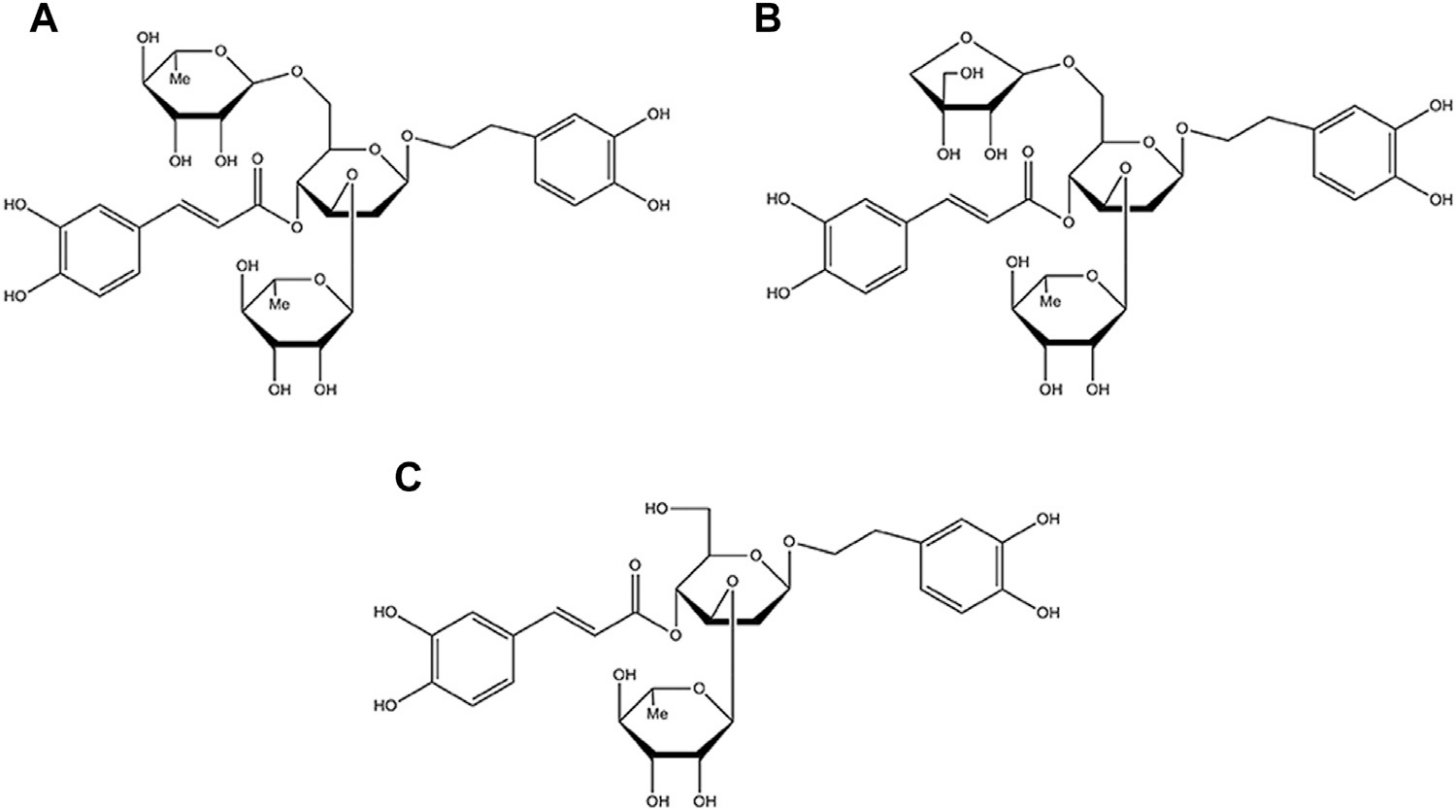
Chemical structure of POL **(A)**, ACT **(B)**, FTB **(C)**.

*Callicarpa kwangtungensis Chun* (CK), a traditional Chinese medicine of *Callicarpa* genus of the Verbenaceae family, has been used for activating blood circulation, promoting *Qi* circulation and relieving pain in traditional Chinese medicine theory (Tu et al., 2013; Sun et al., 2020). Chemically, phenylethanol glycosides (PGs) including poliumoside (POL), acteoside (ACT), and forsythiaside B (FTB) are the major active components of CK (the chemical structure of POL, ACT, FTB are shown in Figure 1) (Shi et al., 2013; Cai et al., 2014). Some of PGs possessed extensive pharmacological activities involving analgesic, antioxidant, anti-inflammatory, anticancer, liver protective, immune regulation and antiviral activities (Delazar et al., 2005; Yang et al., 2020). Therefore, this study was designed to explore the protective effects of POL, ACT, and FTB against injury of ALI cell model induced by TNF-α, and comparatively investigated the potential mechanisms. Additionally, we next scrutinized the roles of POL, ACT, and FTB on the NF-κB and Nrf2 pathways.

## Materials and Methods

### Drugs and Chemicals

Poliumoside, acteoside, and forsythiaside B (all purities more than 98%) were supplied by the Guangdong Provincial Key Laboratory of New Drug Development and Research of Chinese Medicine, Guangzhou University of Chinese Medicine (Guangzhou, China). They were dissolved in sterile distilled water. Human TNF-α was acquired from Sigma-Aldrich (St. Louis, MO, United States). Enzyme-linked immunosorbent assay (ELISA) kits for human IL-1β, IL-6, and IL-8 were purchased from Shanghai Enzyme-linked Biotechnology Co., Ltd. (Shanghai, China). ROS Assay kit was supplied by the NanJing JianCheng Bioengineering Institute (NanJing, China). Annexin V–fluorescein isothiocyanate apoptotic kits were purchased from BestBio (Shanghai, China). The total, nuclear and cytoplasmic protein extraction kits were obtained from Keygene Biotech Co., Ltd. (Nanjing, China). Antibodies for NF-κB p65, *p*-IκBα, Nrf2, Keap1, H3, and *β*-actin were supplied by Affinity Biosciences (Cincinnati, OH, United States). All horseradish peroxidase (HRP)-conjugated secondary antibodies were purchased from EarthOx, LLC (CA, United States). siRNA was offered by RiboBio Co., Ltd. (Guangzhou, China), Lipofectamine® 2000 Reagent was obtained from Invitrogen Co., Ltd. (Carlsbad, CA, United States). Opti-MEM® I Reduced Serum Medium was offered by Gibco Co., Ltd.

### Cell Culture

A549 cells derived from Cell Bioscience Inc. (Shanghai, China). They were maintained in Roswell Park Memorial Institute-1640 (RPMI-1640) medium containing 10% FBS and 1% penicillin-streptomycin. A549 cells were incubated at 37°C in 90% humidified atmosphere with 5% CO_2_.

### Cell Toxicity Test

A549 cells were plated at a density of 4 ×10^4^ cells/well in 96-well plates and were cultured overnight. Then A549 cells were treated with or without 1, 10, 20, 40, 80, and 160 μM of POL, ACT, and FTB for 24 and 48 h, and then 20 μl MTT was added to each well. Next, we removed the culture medium and added 150 μL DMSO to each well after the cells were cultured for 4 h. Finally, the cell viability was calculated through measuring the optical density (OD) at 490 nm with an automated micro-plate reader.

### Establishment of Acute Lung Injury Model in A549 Cells

A549 cells were taken out of the liquid nitrogen for recovery and were subcultured when approximately 80–90% confluent. According to previous experiments, 10 ng/ml TNF-α was the optimal concentration for modeling because it was the smallest effective dose that caused the smallest cell damage (Yang et al., 2018). Therefore, A549 cells were stimulated with 10 ng/ml of TNF-α for establishing acute lung injury model *in vitro*. After 24 h, cells grown in 96-well plates (4 ×10^4^ cells/well) were incubated for 24 h or 48 h with or without different concentrations of POL (10, 20, and 40 μM). In addition, A549 cells were treated with different concentrations of ACT (10, 20, and 40 μM) and FTB (10, 20, and 40 μM) separately as described above. Finally, we collected cell culture fluid and cells for the following experiments.

### siRNA Transfection

The small interfering RNAs (siRNAs) and relative negative control siRNAs of Nrf2 and NF-κB p65 were purchased from RiboBio Company (Guangzhou, China). The use of recombinant RNA (siRNA) was approved for use at our institution. According to the results of PCR and western blot for screening the effective siRNAs of Nrf2 and NF-κB p65, the most efficient siRNAs were determined by our group. The sequences of si-h-RELA gene for the present experiment were as follows: si-RELA-3: CCA​TCA​ACT​ATG​ATG​AGT​T; si-NFE2LE-3: CCA​AAG​AGC​AGT​TCA​ATG​A. A549 cells were transfected with Lipofectamine 2000 Reagent (Invitrogen, CA, United States) using a standard transfection protocol at a final siRNA concentration of 40 nM as previously described (Yang et al., 2018) when their density reached approximately 80% confluence. Finally, the cells were collected and used for further investigations.

### Apoptosis Analysis

Cellular apoptosis was evaluated by using Annexin V-FITC apoptosis detection kit (BestBio Biotechnologies, Shanghai, China). Firstly, A549 cells treated with different drugs (POL, ACT, FTB) were digested with 0.25% trypsin and then resuspended in wash buffer. Cell suspension was harvested for the following experiments. Then the cells were washed with ice-cold phosphate buffer saline (PBS) three times and immediately fixed using the appropriate amount of Binding Buffer for resuspending the cells. After fixation, cells were added with 5 μl of Annexin V-fluorescein isothiocyanate (FITC) and 5 μl of propidium iodide (PI) before incubating in the dark for 15 min. Detection of cell apoptosis by using the flow cytometric analysis (BD Biosciences, San Jose, CA, United States).

### ROS Activity Assay

Cells were treated as described in the apoptosis analysis section and used in ROS activity assay. Then the cells were mixed with 10 μM DCFH-DA and incubated for 30 min at 37°C, protected from light. The intensity of fluorescence was subsequently observed on a fluorescence microscope (Nikon Eclipse, Japan).

### Determination of IL-1β, IL-6, and IL-8

Cells were treated as described in the apoptosis analysis section and the contents of inflammatory factors IL-1β, IL-6, and IL-8 were detected using Elisa kits following the manufacturer’s protocol.

### Real-Time Quantitative Polymerase Chain Reaction (RT-PCR) Analysis

Total RNA was extracted from cells by TRIzol reagent (Invitrogen, Shanghai, China) and its purity was determined using NanoDrop 2000 (Thermo, American). The cDNA was synthetized with reverse transcription kit (Applied Biosystems, Branch burg, NJ, United States) and reverse transcription was performed according to the manufacturer’s recommendations. PCR amplification reaction conditions: stage 1: 95°C for 30 s; stage 2: 40 cycles at 95°C for 10 s and 60°C for 30 s; stage 3: 95°C for 15 s, 60°C for 60 s, and 95°C for 15 s. The primers synthesized by Sangon Biotech (Shanghai) company for RT-PCR are listed in Supplementary Table S1.

### Western Blot Analysis

The total, nuclear and cytoplasmic protein were extracted from cells separately using their corresponding extraction kits. And the protein concentration was measured by the BCA protein assay kit (Bio-Rad, Hercules, CA). Then whole cell lysates were separated by 10% SDS-PAGE, transferred onto polyvinylidene fluoride (PVDF) membranes and treated with 5% skimmed milk for 1 h. Then the membrane was washed with TBST 3 times for 10 min each time, and incubated with appropriate primary antibodies against NF-κB p65 (1:1,000), *p*-IκBα (1:1,000), Nrf2 (1:1,000), Keap1 (1:1,000), H3 (1:1,000), and *β*-actin (1:1,000) overnight at 4°C. Subsequently, the membrane was washed with TBST 3 times, 10 min each time. The TBST was removed and the membrane was incubated with secondary antibody for 1 h, and washed with TBST twice for 10 min. The protein signals were detected by ECL reagent (Bio-Rad, Hercules, CA) and the gray value of the target band was analyzed using ImageJ software. Concomitantly, *β*-actin and H3 were measured as a loading control.

### Statistical Analysis

All data were analyzed using SPSS 26.0 software and expressed as mean ± standard deviation. Data were evaluated by One-way ANOVA followed by LSD or Dunnett’*t* test for comparisons. Of those, *p* < 0.05 or *p* < 0.01 were considered as statistically significant.

## Results

### Cytotoxicities of POL, ACT, and FTB in A549 Cells

**FIGURE 2:**
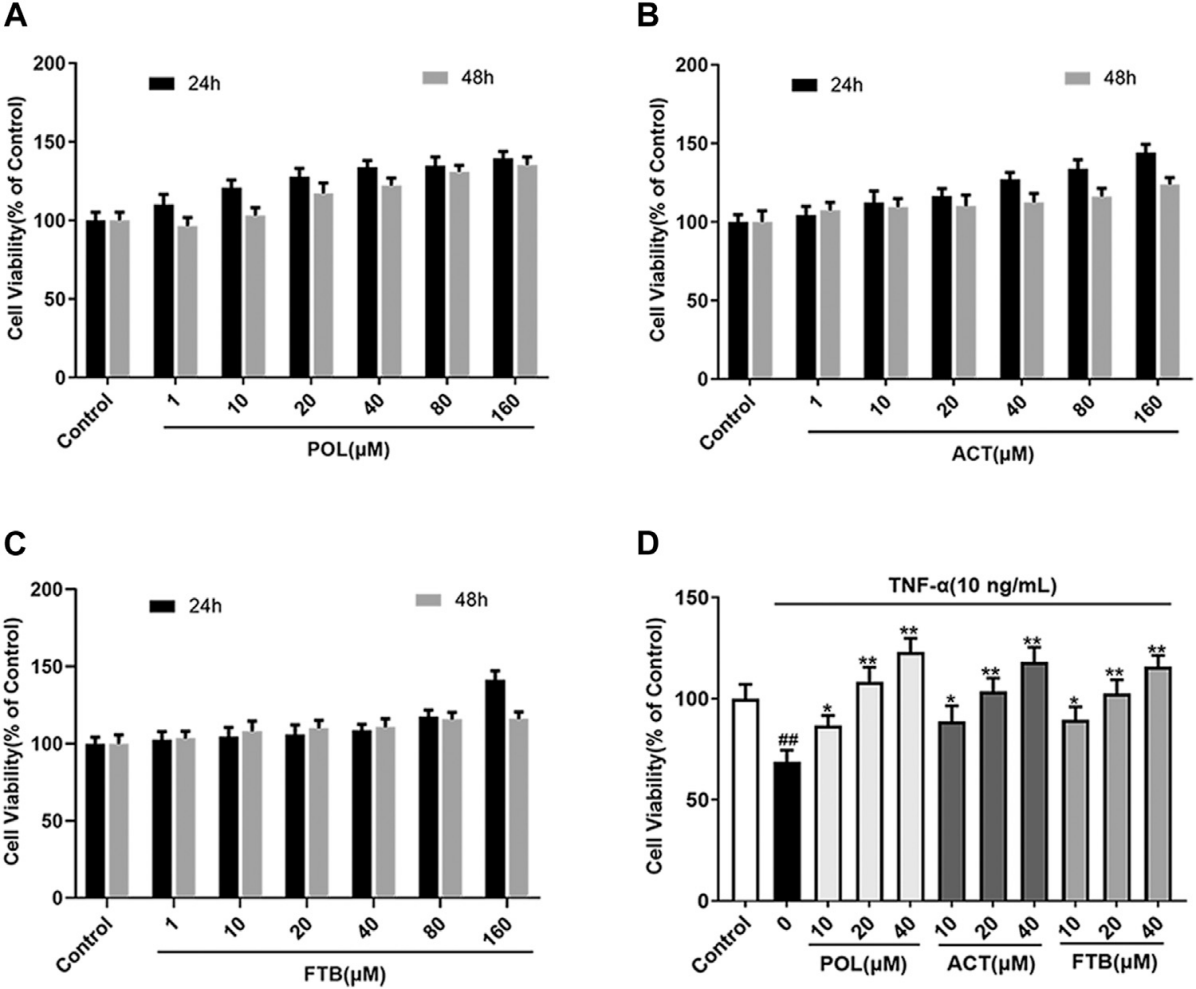
Effects of POL, ACT, and FTB on A549 cells. **(A–C)** Effects of treatment with POL, ACT, FTB (1, 10, 20, 40, 80, and 160 μM) for 24 and 48 h on the viability of A549 cells. **(D)** Cell viability was measured by stimulating with TNF-α (10 ng/ml) for 24 h, and then treated with POL, ACT, and FTB (10, 20, and 40 μM) for 24 h. Values represent the means ± SD (*n* = 6). ^##^ *p* < 0.01 vs control; ^*^ *p* < 0.05, **p < 0.01 vs TNF-α.

The effects of POL, ACT, and FTB on cell growth were shown in Figure 2. Compared with control group, POL, ACT, and FTB at the concentrations 1 μM up to 160 μM didn’t show significant cytotoxicity to A549 cells when incubated for 24 h (Figures 2A–C). For the above three compounds, no significant difference was found between treatment of 24 and 48 h in cell viability. As shown in Figure 2D, the cell viability in the TNF-α group was obviously lower than in the control group. Meanwhile, POL (10, 20, and 40 μM) increased cell viability dose-dependently when compared with TNF-α group, especially in the high-dose group. Similarly, 10, 20, and 40 μM of ACT and FTB also had protective effects against TNF-α induced cell damage in the A549 cells. Based on the above results, the concentrations of 10, 20, and 40 μM for POL, ACT, and FTB were used for the following experiments.

### Effects of POL, ACT, and FTB on Cell Apoptosis Rate, ROS Levels, Apoptosis-Related Genes and Inflammatory Factors

**FIGURE 3:**
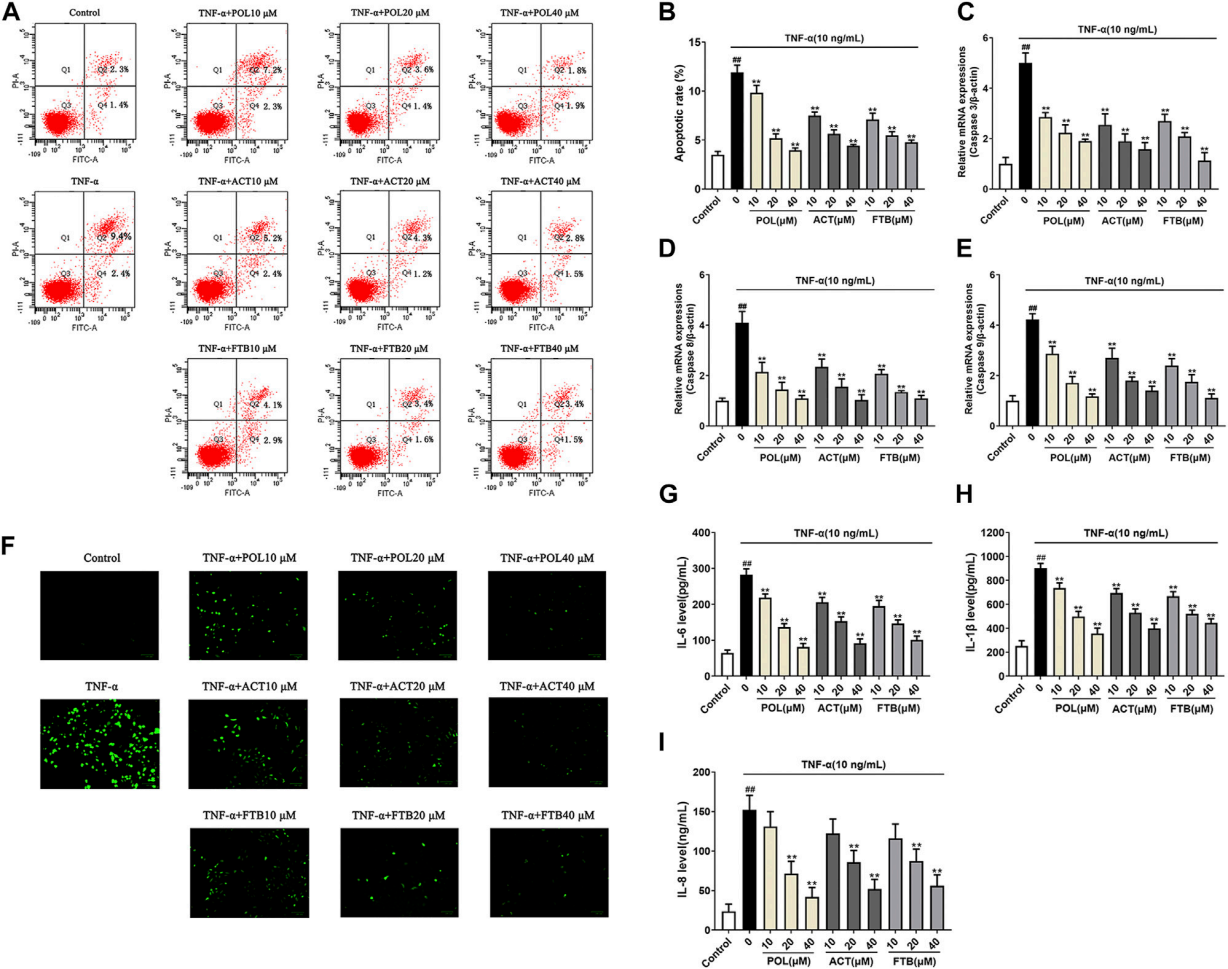
Effects of POL, ACT, and FTB on cell apoptosis rate, ROS levels, apoptosis-related genes and inflammatory factors **(A,B)** Effects of POL, ACT, and FTB on TNF-α-induced apoptosis were evaluated using flow cytometry **(C–E)** Relative mRNA expression levels of Caspase 3, Caspase 8, and Caspase 9 **(F)** Effects of POL, ACT, and FTB on TNF-α-induced ROS levels were observed using a fluorescence microscope. Scale bar indicates 100 μm **(G–I)** Effects of POL, ACT, and FTB on TNF-α-induced expression levels of IL-8, IL-1β, and IL-6 were determined using ELISA kits. Values represent the means ± SD (*n* = 3-6). ^##^ *p* < 0.01 vs control, **p < 0.01 vs TNF-α.

As shown in Figures 3A,F, the TNF-α alone group significantly increased the apoptosis rate of cell and ROS levels compared with the control group (*p* < 0.01). After 24 h of treatment with POL (10, 20, and 40 μM), there was a significant reduction in the apoptosis rate of cell and ROS levels in A549 cells induced by TNF-α (all *p* < 0.01). At the same time, the inhibitory effects of ACT (10, 20, and 40 μM) and FTB (10, 20, and 40 μM) on TNF-α-induced injury in A549 cells were similar to POL. Consistent with the protective effects of POL, ACT, and FTB, the levels of Caspase-3, Caspase 8, and Caspase 9 were significantly decreased in three treatment groups (POL, ACT, and FTB) (Figures 3C–E). The ELISA results of IL-8, IL-6, and IL-1β were shown in Figures 3G–I. Compared to TNF-α alone group, a marked decrease was seen in the expression levels of IL-8, IL-6, and IL-1β after treatment with POL (10, 20, and 40 μM), ACT (10, 20, and 40 μM), and FTB (10, 20, and 40 μM). All these data suggested that treatment with POL, ACT, and FTB not only suppressed inflammation but also alleviated TNF-α-induced injury.

### Effects of POL, ACT, and FTB on mRNA Expression of Genes Related to Cell Inflammation and Oxidative Stress in Cells

**FIGURE 4:**
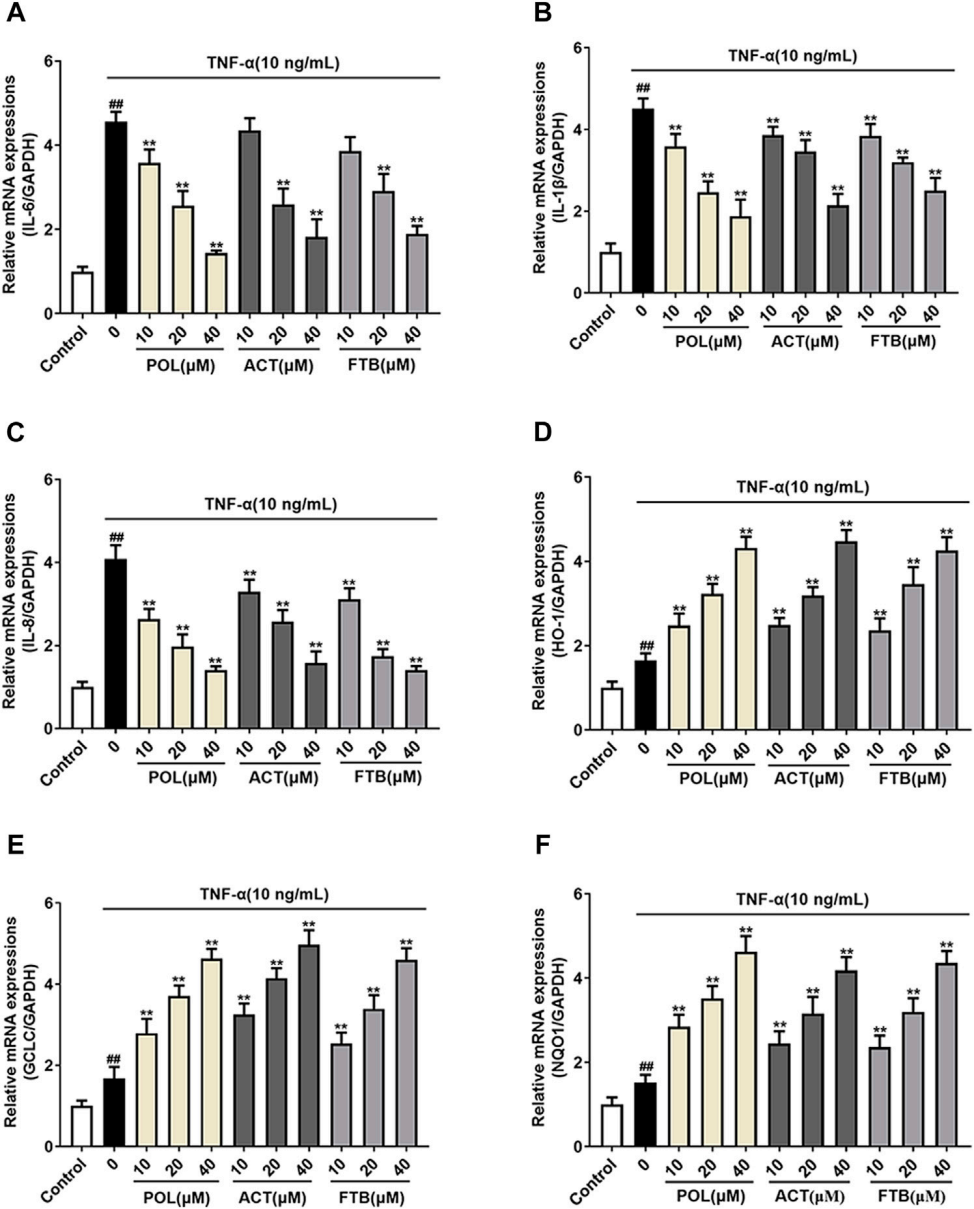
Effects of POL, ACT, and FTB on TNF-α-induced cell inflammation and oxidative stress **(A–F)** Relative mRNA expression levels of NQO1, GCLC, HO-1, IL-1β, IL-6, and IL-8. Values represent the means ± SD (*n* = 6). ^##^ *p* < 0.01 vs control; **p < 0.01 vs TNF-α.

To further study whether the protective effects of POL, ACT, and FTB on A549 cells were associated with anti-inflammatory and antioxidant effects, we examined the mRNA expression of pro-inflammatory cytokines (IL-6, IL-1β, and IL-8) and anti-oxidant factors (HO-1, GCLC, and NQO1) (Figure 4). Compared with the control group, TNF-α stimulated cells to produce over-expression of pro-inflammatory cytokines. On the contrary, cells that were treated with POL (10, 20, and 40 μM) for 24 h significantly down-regulated the expression of pro-inflammatory cytokines (Figures 4A–C). In addition, ACT (10, 20, and 40 μM) and FTB (10, 20, and 40 μM) had the same positive effects on cells with that of POL. As shown in Figures 4D-F, it was observed that the levels of anti-oxidant factors (HO-1, GCLC, and NQO1) were enhanced in TNF-α group when compared with control group. While compared with the TNF-alone group, administration with POL (10, 20, and 40 μM) more significantly enhanced the expression of anti-oxidant factors (HO-1, GCLC, and NQO1) (all *p* < 0.01). In addition, ACT (10, 20, and 40 μM) and FTB (10, 20, 40 μM) could also significantly upregulate the expression of anti-oxidant factors (HO-1, GCLC, and NQO1) (all *p* < 0.01) when compared with TNF-α group.

### Effects of POL, ACT, and FTB on NF-κB Pathway and Keap1-Nrf2 Pathway

**FIGURE 5:**
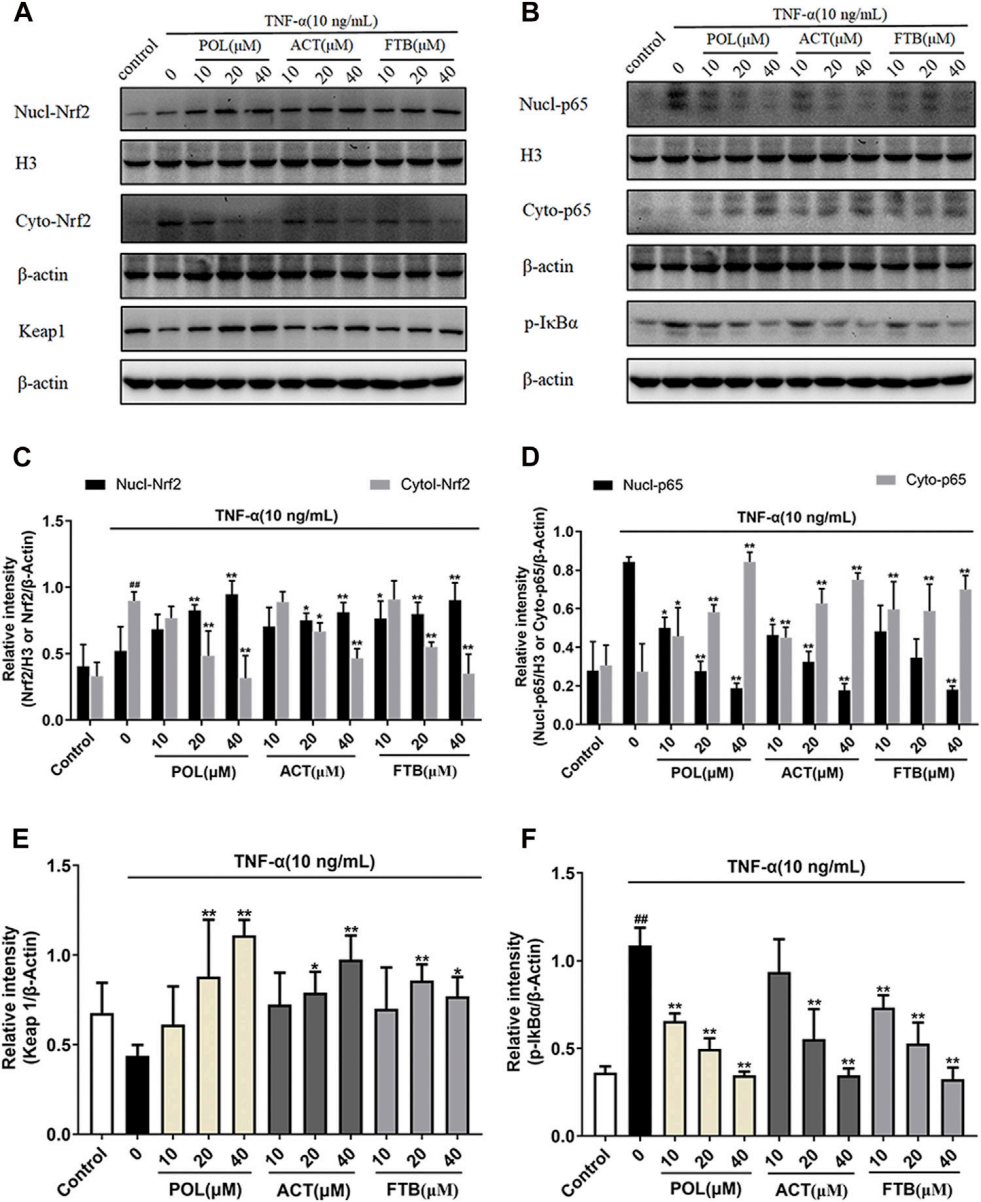
Effects of POL, ACT, and FTB on Keap1-Nrf2 and NF-κB p65 signaling pathways in TNF-α-induced cell injury in A549 cells **(A,B)** Western blot analyses of nucl-Nrf2, cyto-Nrf2, Keap1, nucl-p65, cyto-p65, and *p*-IκBα **(C–F)** Intensity of Keap1, cyto-Nrf2, *p*-IκBα, and cyto-p65 relative to *β*-actin. Intensity of nucl-Nrf2, nucl-p65 relative to H3. Values represent the means ± SD (*n* = 3). ^##^ *p* < 0.01 vs control; ^*^ *p* < 0.05, **p < 0.01 vs TNF-α.

The western blot results suggested that the levels of cytoplasmic Nrf2, nuclear NF-κB p65 and *p*-IκBα increased significantly in the TNF-α alone group compared with the control group (Figures 5A,B). Compared with the TNF-α alone group, all tested dosages of POL, ACT, and FTB could significantly increase the expression levels of nuclear-Nrf2, cyto-p65, Keap1 (Figures 5C–F). Conversely, the expression levels of cyto-Nrf2, nuclear-p65, *p*-IκBα were dramatically reduced in above comparison (Figures 5C–F). These results demonstrated that POL, ACT, and FTB alleviated the TNF-α induced cell damage by modulating the NF-κB pathway and Nrf2 pathway concomitantly.

### Effects of POL, ACT, and FTB on Cell Injury After siRNA Knockdown of Nrf2 and NF-κB p65 Genes in TNF-α Induced A549 Cells

**FIGURE 6:**
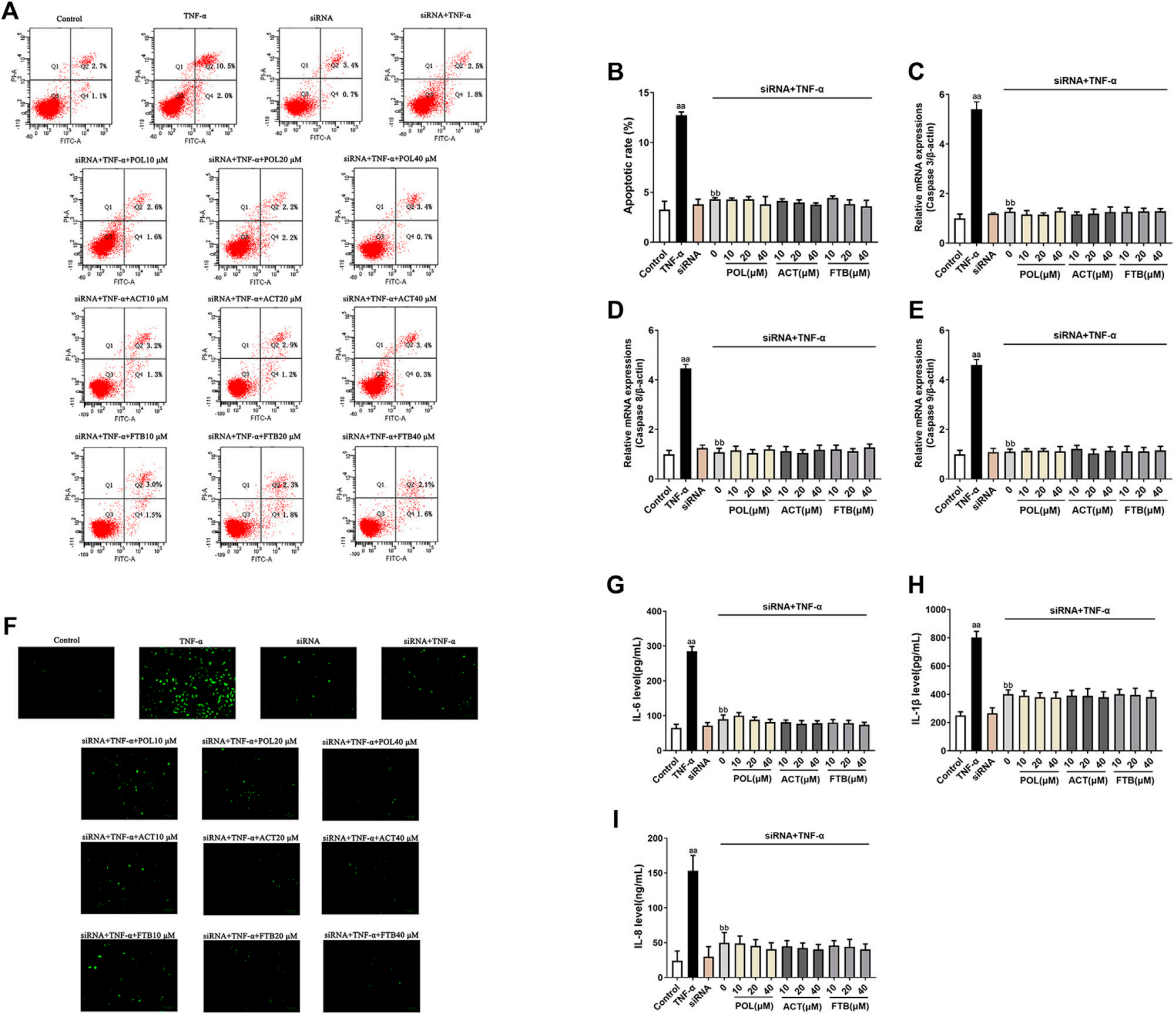
Effects of POL, ACT, and FTB on TNF-α-induced apoptosis, ROS levels, and expression levels of cytokines following Nrf2 and NF-κB p65 siRNA knockdown. **(A,B)** Effects of POL, ACT, and FTB on TNF-α-induced apoptosis following Nrf2 and NF-κB p65 siRNA knockdown **(C–E)** Relative mRNA expression levels of Caspase 3, Caspase 8, and Caspase 9. **(F)** Effects of POL, ACT, and FTB on the TNF-α-induced ROS level following Nrf2 and NF-κB p65 siRNA knockdown. Scale bar indicates 100 μm **(G–I)** Effects of POL, ACT, and FTB on TNF-α-induced IL-1β, IL-8, and IL-6 levels following Nrf2 and NF-κB p65 siRNA knockdown. Values represent means ± SD (*n* = 3-6). ^aa^ *P* < 0.01 vs control, ^bb^ *P* < 0.01 vs TNF-α.

To analyze whether the protective effects of POL, ACT, and FTB against injury was mediated by the NF-κB and Nrf2 pathways, we transfected cells with siRNA designed to knockdown Nrf2 and NF-κB p65 and then determined the expression of ALI-related genes. As demonstrated in Figure 6, the apoptosis rate, ROS levels, the expression of apoptosis-related genes (Caspase 3, Caspase 8, and Caspase 9) and inflammatory factors (IL-8, IL-1β, and IL-6) of A549 cells induced by TNF-α alone were significantly increased (*p* < 0.01) as compared to the control group. No effect on the apoptosis rate, ROS levels, the expression of apoptosis-related genes (Caspase 3, Caspase 8, and Caspase 9) and inflammatory factors including IL-8, IL-1β, and IL-6 of A549 cells occurred siRNA alone group with the control group. It suggested that siRNA chosen has no damage on A549 cells. Moreover, we found that the apoptosis rate, ROS levels, the levels of Caspase 3, Caspase 8, Caspase 9, IL-8, IL-1β, and IL-6 decreased markedly after the administration of siRNA+TNF-α than TNF-α alone (all *p* < 0.01). Interestingly, siRNA+TNF-α POL (10, 20, and 40 μM) showed no significant differences on all above indices with siRNA+TNF-α (all *p* < 0.01). Furthermore, compared with siRNA+TNF-α, no significant changes were observed in siRNA+TNF-α ACT (10, 20, and 40 μM) and siRNA+TNF-α FTB (10, 20, and 40 μM). Together these results suggested that the protective effects of POL, ACT, and FTB against oxidative damage and inflammatory response induced by TNF-α were abolished after knockdown of Nrf2 and NF-κB p65 pathways.

## Discussion

The essential role of inflammatory responses in health and disease has long been recognized. Local acute inflammation represents a protective means for host resistance to tissue injury resulting from microbial infection (Serhan and Petasis, 2011). ALI is a serious disease characterized by acute inflammation, with a high mortality rate (Ortiz-Diaz et al., 2013). Numerous inflammatory diseases are related to TNF-α (Popa et al., 2007). TNF-α has been confirmed that it was involved in the pathophysiology of a number of inflammatory lung diseases, such as asthma, chronic bronchitis and ARDS (Lee et al., 2013). And TNF-α was considered to play an important role in ALI. For this reason, it was commonly used to establish ALI model *in vitro* in the past years (Yang et al., 2018). Therefore, TNF-α was used to establish the ALI model *in vitro* in this study.

NF-κB signaling pathway is a considerable transduction pathway involved in cellular inflammation and immune response which is viewed as a key therapeutic target (Zhu et al., 2015). Several studies indicated that NF-κB p65 of cytoplasm always binds to the inhibitory protein IκBs at rest in normal circumstances. They dissociate rapidly and NF-κB p65 is transferred into the cell nucleus for expression when stimulated by different irritation of outside world (Hayden and Ghosh, 2008). Moreover, pro-inflammatory cytokines mediated inflammation in ALI closely, including TNF-α, IL-1β, IL-6, and IL-8 (Goodman et al., 2003). Previous studies demonstrated that activation of NF-κB caused robust up-regulation of the expression of IL-1β, IL-6, and IL-8 on ALI model (Chang et al., 2015). A previous study showed that NF-κB p65 was activated by ROS, and thereby tightly linked oxidative stress with inflammation (Moon et al., 2011). When stimulated by ROS, NF-κB p65 was isolated from IκBα and metastasized to the nucleus rapidly, which caused a massive expression of anti-inflammatory cytokines that led to expressing significant amounts of ROS again. In this study, POL, ACT, and FTB potently suppressed NF-κB p65 and IκBα phosphorylation, protecting A549 cells from inflammation. And we found that the levels of IL-1β, IL-6, IL-8, nucl-p65, and *p*-IκBα of three treatment groups (POL, ACT, and FTB) were markedly lower than those of TNF-α alone group. Notably, the decline was especially pronounced at higher doses. In our study, we found that FTB at a dose of 10 μM brought on the largest decrease in the expression of IL-1β, IL-6, IL-8 and protein expression of nucl-p65. However, POL showed the most striking differences in the medium and high dose group (20, 40 μM) in all active treatments. Given these facts, we reasoned that POL had strongest anti-inflammatory activity and it was more effective on mediating the NF-κB pathway. These observations indicated that POL, ACT, and FTB were beneficial in reducing pulmonary inflammation through suppressing NF-κB activation and blocking IκBα degradation, leading to suppression of proinflammatory gene programs and lower expression of IL-1β, IL-6, and IL-8. Moreover, POL was the most effective ingredient among them in protecting against TNF-α induced cellular inflammation.

It is generally assumed that oxidative stress is associated with a disbalance between the generation of ROS and the biologic scavenger system (Pratheeshkumar et al., 2014). Oxidative stress is involved in the cell death process and oxidative damage has been demonstrated to be one of the underlying mechanisms that contribute to ALI (Guo and Ward, 2007). It is well established that Nrf2 is sensitive to oxidative stress which is considered as a major contributor to most ALI (Reddy et al., 2009; Singh et al., 2011). The Keap1-Nrf2 signaling pathway is an indispensable defensive transduction pathway for cells to resist oxidative stress and maintain a balance of oxidation. Accumulating evidence suggests that activating the Nrf2 pathway could greatly reduce oxidative damage and cell apoptosis in ALI. Under normal physiological conditions, Keap1 and Nrf2 are in a resting state. When stimulated by ROS or electrophiles, decoupled Nrf2 transfers into the nucleus to bind to ARE, and thus initiates the transcription of downstream antioxidant protein genes (Cho et al., 2006; Jiang et al., 2016). Among these, a series of anti-oxidative enzymes (including HO-1, NQO1, and GCLC) are involved in the antioxidant process of body, which could inhibit the oxidative stress response of ROS to prevent cells from oxidative damage (Haines et al., 2012; Han et al., 2012). Experimental evidence suggested that POL, ACT, and FTB not only increased the expression of anti-oxidative enzymes (including HO-1, NQO1, and GCLC), but also obviously increased nuclear-Nrf2 and Keap1. Furthermore, POL, ACT, and FTB obviously decreased the level of inflammatory cytokines, as well as attenuated ROS activity and cell apoptosis. In our study, POL, ACT, and FTB significantly improved the mRNA expression of HO-1 and GCLC, without significant between-group differences. As for NQO1, POL was more effective than ACT and FTB in the same dosage. In addition a high dose of POL showed greater increase in the levels of nuclear-Nrf2, cyto-p65, and Keap1. Therefore, we concluded that POL had the best anti-oxidant effect among them. Compared with ACT and FTB, POL was more effective on mediating Keap1-Nrf2 pathway. Above results revealed that the activation of Keap1-Nrf2 pathway might play a key role in TNF-α triggering ALI.

Indeed, a large number of inflammatory diseases are closely related to crosstalk between the NF-κB and Keap1-Nrf2 pathways (Yerra et al., 2013; Wardyn et al., 2015). Bak et al. found that HO-1, a Nrf2 target gene, worked on the process of Nrf2-mediated NF-κB inhibition (Bak et al., 2017). It was reported that ethanol extract of Alisma Rhizoma reduced ALI *via* suppressing NF-κB and activating Nrf2 (Han et al., 2013). Thimmulappa et al. found that knocking out of Nrf2 gene in the experimental animal model led to an increase in NF-κB activity and the incidence of ALI animals. The degree of lung injury in the Nrf2 knockdown group was more serious compared with that in the normal group (Thimmulappa et al., 2006). Several previous studies demonstrated that some herbs and active ingredients had also been used to ameliorate ALI through activation of Nrf2 and suppression of NF-κB (Lyu et al., 2012; Lelli et al., 2017; Lv et al., 2017; Yang et al., 2018). The Nrf2 pathway interacted with the NF-κB pathway, they influenced each other, thus leading to oxidative stress, inflammation, apoptosis, and cell damage. Also, they were involved in a multitude of cellular processes including oxidative stress, inflammation, immune responses and cell apoptosis.

In view of the fact that ALI is a multi-step process that can be activated by various factors, especially oxidative stress and inflammatory damage. In current study, POL could significantly up-regulate the expression levels of nucl-Nrf2, cyto-p65, Keap1. Conversely, they significantly down-regulated the expression levels of cyto-Nrf2, nucl-p65, *p*-IκBα. Besides, POL, ACT, and FTB performed similarly in improving inflammation and oxidative stress. Our data suggested that POL, ACT, and FTB might play anti-inflammatory and antioxidant role in reducing cell damage by regulating the NF-κB pathway and Keap1-Nrf2 pathway, inhibiting the phosphorylation levels of IκBα and nuclear p65, and enhancing the expression of Nrf2 and Keap1. In addition, we performed specific gene ablation to further validate whether POL, ACT, and FTB exert anti-inflammatory and anti-oxidant effects by mediating the NF-κB and Nrf2 pathways. Subsequently, we observed the apoptosis rate, ROS levels, the levels of inflammatory factors (IL-8, IL-1β, and IL-6) and apoptosis-related genes (Caspase 3, Caspase 8, and Caspase 9) following siRNA transfection. Interestingly, no statistical difference was observed in all above indices. The above results showed that the mechanism of Nrf2 and NF-kB p65 siRNA downregulation didn’t affect POL, ACT, and FTB effects. It suggested that Nrf2/NF-κB p65 signaling pathways have been implicated in the anti-inflammatory and anti-oxidant effects of POL, ACT, and FTB in preventing TNF-α-induced A549 cell damage.

Together with the above findings, these results suggested that considerable therapeutic benefits of POL, ACT, and FTB could be expected to improve ALI care efficiently *in vitro*. Among these ingredients, POL possessed the most effective anti-inflammatory and antioxidant properties. For this reason, POL could be further studied to treat ALI. More pharmacological and further medical research experiments are needed. At present, some of these initiatives are already underway in our research group.

## Conclusion

Overall, our study indicated that POL, ACT, and FTB alleviated oxidative damage and lung inflammation in TNF-α-induced ALI cell model through activating Nrf2 pathway and suppressing NF-κB pathway. The results of this study also exhibited that POL achieved similar therapeutic effect as compared to ACT and FTB, and superior to them, which showed that POL might have the potential to be further developed into a promising therapeutic agent for the treatment of ALI. The present research results will hopefully serve as beneficial feedback information for the development of adjuvant drugs and treatment of ALI.

## Data Availability

The raw data supporting the conclusions of this article will be made available by the authors, without undue reservation.
