# Supplementary material for: Phenylethanoid Glycosides From Callicarpa kwangtungensis Chun Attenuate TNF-α-Induced Cell Damage by Inhibiting NF-κB Pathway and Enhancing Nrf2 Pathway in A549 Cells
Source: Front Pharmacol. 2021 Jul 7;12:693983. doi: 10.3389/fphar.2021.693983 (PMC8293607; doi:10.3389/fphar.2021.693983)

Supplementary Materials

# Supplementary Table

Table 1. Primers sequences used for quantitative PCR.

| **Gene name** |  | **Primer (5′-3′)** |
| --- | --- | --- |
| **HO-1** | Forward  Reverse | AAGACTGCGTTCCTGCTCAAC  AAAGCCCTACAGCAACTGTCG |
| **NQO-1** | Forward  Reverse | GAAGAGCACTGATCGTACTGGC  GGATACTGAAAGTTCGCAGGG |
| **GCLC** | Forward  Reverse | GGAGGAAACCAAGCGCCAT  CTTGACGGCGTGGTAGATGT |
| **IL-1β** | Forward  Reverse | AAGCTGATGGCCCTAAACAG  AGGTGCATCGTGCACATAAG |
| **IL-6** | Forward  Reverse | TCCACAAGCGCCTTCGGTCCAGTTG  AGAGGTGAGTGGCTGTCTGTGGG |
| **IL-8** | Forward  Reverse | CTGGCCGTGGCTCTCTCTTG  CCTTGGCAAAACTGCACCTT |
| **GAPDH** | Forward  Reverse | TGTGGGCATCAATGGATTTGG  ACACCATGTATTCCGGGTCAAT |
| **Caspase 3** | Forward  Reverse | TTGAGACAGACAGTGGTGTTGATGATG  TGGCACAAAGCGACTGGATGAAC |
| **Caspase 8** | Forward  Reverse | AGAAGTGAGCAGATCAGAATTGAGGTC  ATATCCAGCAGGTTCATGTCATCATCC |
| **Caspase 9** | Forward  Reverse | AAGAGCACCGACATCACCAAATCC  GACCAGAGATTCGCAAACCAGAGG |
| **β-actin** | Forward  Reverse | CCTGGCACCCAGCACAAT  GGGCCGGACTCGTCATAC |

# Supplementary Figures

- 1. **Supplementary Figures of Western blot**

### β-actin


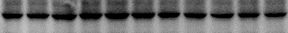


### H3


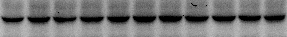

Supplement: Supplementary file 1 [file DataSheet2.docx]
