# Supplementary material for: Phenylethanoid Glycosides From Callicarpa kwangtungensis Chun Attenuate TNF-α-Induced Cell Damage by Inhibiting NF-κB Pathway and Enhancing Nrf2 Pathway in A549 Cells
Source: Front Pharmacol. 2021 Jul 7;12:693983. doi: 10.3389/fphar.2021.693983 (PMC8293607; doi:10.3389/fphar.2021.693983)
Supplement: Supplementary file 2 [file DataSheet1.docx]

Supplementary Material

# Supplementary Data

1.1 Methods

The A549 cell line was obtained from American Type Culture Collection (ATCC) (Rockville, MD). Cells were cultured in Roswell Park Memorial Institute (RPMI) 1640 Medium supplemented with 10% fetal bovine serum and 1% Penicillin-Streptomycin. The cells were incubated in a humidified incubator with 5% CO2 at 37℃.

1.2 siRNA preparation and transfection

The si-h-RELA gene-specific sequences are as follows: si-RELA-1: CCCTGAGCACCATCAACTA, si-RELA-2: GCCCTATCCCTTTACGTCA, si-RELA-3: CCATCAACTATGATGAGTT. The si-h-NFE2L2 gene-specific sequences are as follows: si-NFE2L2-1: GAGAAAGAATTGCCTGTAA, si-NFE2L2-2: GAGACTACCATGGTTCCAA, si-NFE2LE-3: CCAAAGAGCAGTTCAATGA. The negative control siRNA was synthesized by the RiboBio Company (Guangzhou, China). Lipofectamine®2000 Reagent (Invitrogen, Carlsbad, CA, USA) was used as the transfection agent. Transfection of siRNA was carried out according to the manufacturer’s protocol.

Briefly, when the A549 cells confluence about to 80% in six well plates started to transfect. First, 10 μL of Lipofectamine®2000 Reagent was mixed with 240 μL Opti-Mem and incubated 5 minutes at room temperature; 4 μLof siRNA were mixed with 246 μL Opti-Mem incubated 5 minutes at room temperature. Then two mixtures were mixed together for a final volume of 500 μL and the mixture was incubated at room temperature for 20 minutes. Finally, 1.5 mL Opti-Mem added into the mixture and started to transfect. About 5 hours later, changed with normal 1640 medium without PS, and the final siRNA concentration was 40 nM.

1.3 Real-time quantitative PCR

Total RNA was isolated from A549 cells using the TRIzol reagents (Invitrogen). Immediately, 2 ug RNA using the HiScript II Reverse Transcriptase Kit to synthetic cDNA. Then applying the SYBR fluorescence probe to PCR-amplified the cDNA, the programs were run at stage 1: 95 ℃ for 30 secs; stage 2: followed by 40 cycles at 95 ℃ for 10 secs and 60 ℃ for 30 secs; stage 3: 95 ℃ for 15 secs, 60℃ for 60 secs and 95 ℃ for 15 secs. GAPDH was used as an internal control. Fold change= 2-ΔΔCt, ΔΔ Ct = (Ct Sample– Ct GAPDH) – (Ct Control– Ct GAPDH). The primers sequences of gene including GAPDH, Nrf2 and NF-κB p65 were synthesized by Invitrogen (Carlsbad, CA, UAS), the siRNA sequences were listed as Table S1.

1.4 Western blot analysis

The cells were washed in pre-cold PBS three times, using Nuclear and Cytoplasmic Protein Extraction Kit (KeyGEN BioTECH) to extract the total protein. Then using the BCA Protein Quantitation Assay Kit (KeyGEN BioTECH) to measure the protein concentrations and unified the loading quantities. Next, separated by 12% SDS polyacrylamide gel electrophoresis (PAGE) and electrophoresis to transfer onto PVDF membranes and washed with TBST twice times. Then blocked for 1 h with PBS containing 5% dried milk powder and incubated overnight at 4°C with primary antibodies. The membranes were then washed in TBST three times, and the appropriate HRP-conjugated secondary antibodies diluted (1:2000) in 5% dried milk incubated 1h, washed with TBST three times and developed.

**Table S1**. Primers used for PCR

| **Gene** | **Forward primer (5′ to 3′)** | **Reverse primer (3′ to 5′)** |
| --- | --- | --- |
| RELA | CCCACGAGCTTGTAGGAAAGG | GGATTCCCAGGTTCTGGAAAC |
| NFE2L2 | TCAGCGACGGAAAGAGTATGA | CCACTGGTTTCTGACTGGATGT |
| GAPDH | TGTGGGCATCAATGGATTTGG | ACACCATGTATTCCGGGTCAAT |

2. Results

2.1 Nrf2 and NF-κB p65 expression are suppressed with Nrf2 and NF-κB p65 siRNA knockdown

RT-PCR analysis suggested that after 24 h transfected with seven pairs of siRNAs, the mRNA expression level of Nrf2 and NF-κB p65 were significantly decreased compared with NC-siRNA (p < 0.05) (Fig. S2 A), respectively. Moreover, the protein levels of Nrf2 and NF-κB p65 in A549 cells were significantly down-regulated by transfected siRNAs for 24 h (Fig. S2 B). According to the results, the most effective siRNAs were used in the subsequent studies.

To further verify the effective of siRNAs, the RELA-siRNA3 and NFE2L2-siRNA3 were co-transfected 24 and 48 hours. As shown in Fig. S3 A and B, RT-PCR and Western blot results showed that theses siRNAs could significantly knockdown the expression level of Nrf2 and NF-κB p65, compared with NC-siRNA (p < 0.01).


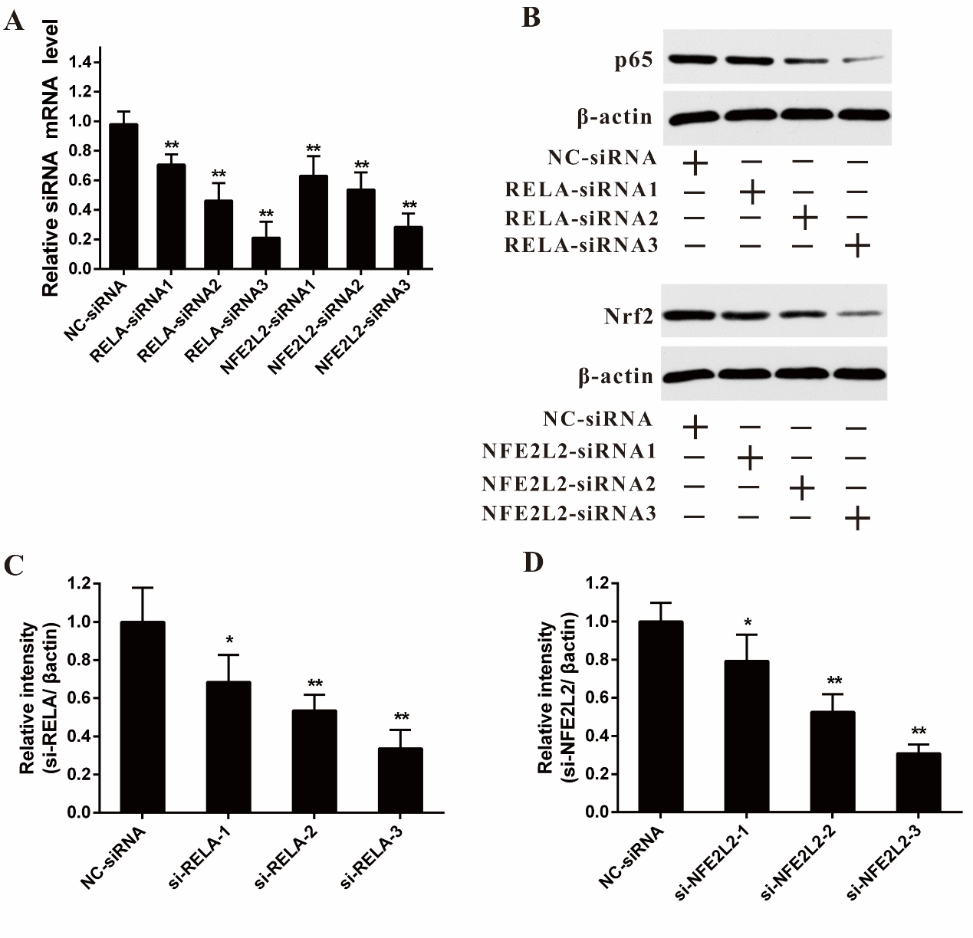


**Fig. S2.** The expressions of Nrf2 and NF-κB p65 were suppressed with Nrf2 and NF-κB p65 siRNA. (A) RT-PCR analysis of relative mRNA levels of Nrf2 and NF-κB p65 for 24 hrs after transfected with seven pairs of siRNAs. (B) Western blot analysis of relative protein levels of Nrf2 and NF-κB p65 for 24 h after transfected. (C-D) Relative intensity of Nrf2 and NF-κB p65 to β-actin. Values represent the means ± SD (n=4). ^*^ p < 0.05, ^**^ p < 0.01 vs. NC-siRNA.


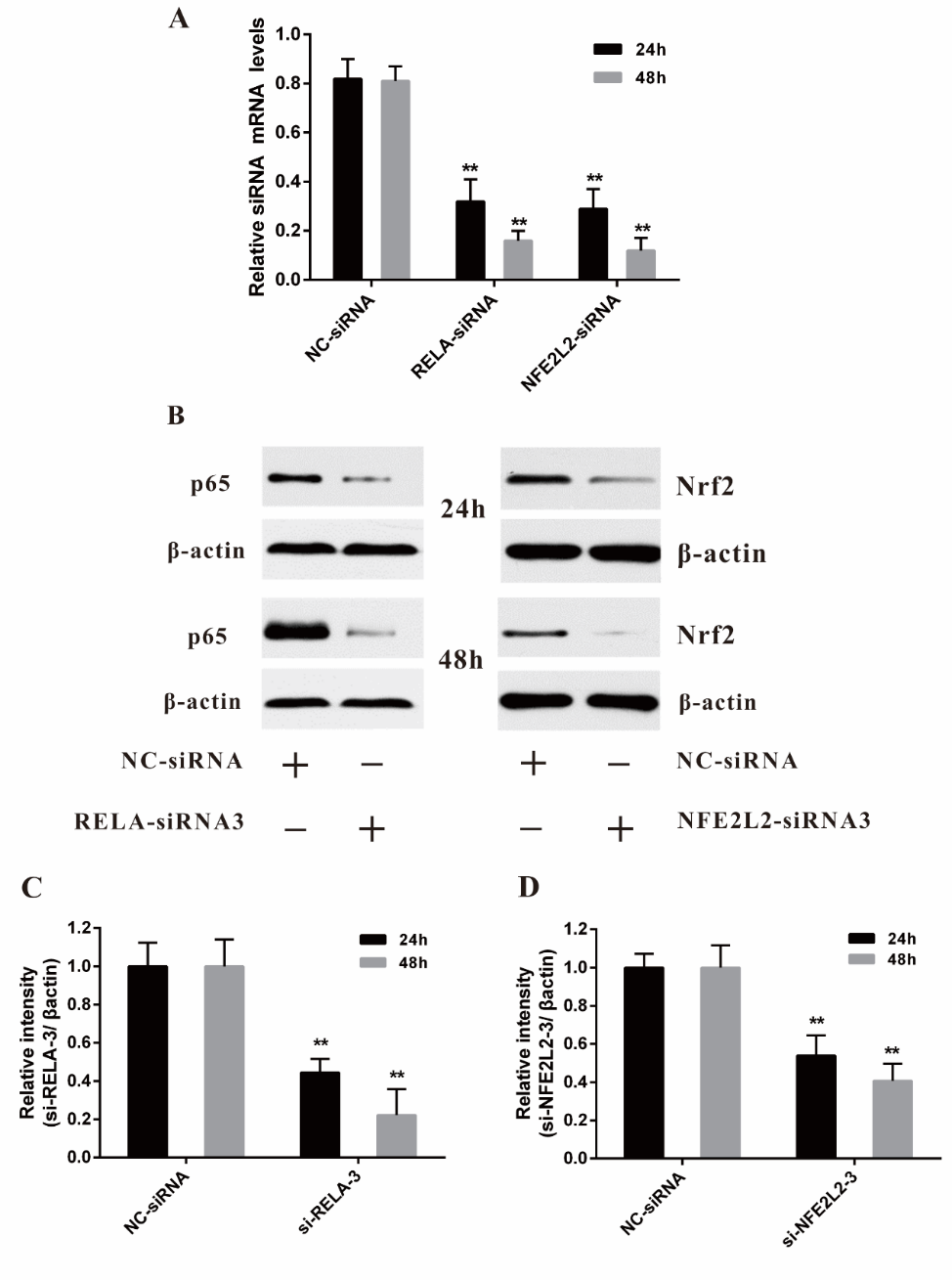


**Fig. S3.** The expressions of Nrf2 and NF-κB p65 were suppressed with Nrf2 and NF-κB p65 siRNA co-transfected 24 and 48 h. (A, B) RT-PCR and western blot analysis of relative mRNA levels of Nrf2 and NF-κB p65 after the relative effectively siRNAs co-transfected 24 and 48 h.(C-D) Relative intensity of Nrf2 and NF-κB p65 to β-actin. Values represent the means ± SD (n=4). ^**^ *p* < 0.01 vs. NC-siRNA.

# Supplementary Figures and Tables

- 1. **Supplementary Figures of Western blot**

### β-actin 42kDa





**Repeat1**





**Repeat2**





**Repeat3**

### cyto-β-actin 42kDa





**Repeat1**





**Repeat2**





**Repeat3**

### H3 15kDa

**

**

**Repeat1**





**Repeat2**





**Repeat3**

### cyto-nrf2 68kDa





**Repeat1**





**Repeat2**





**Repeat3**

### nucl-nrf2 68kDa





**Repeat1**





**Repeat2**





**Repeat3**

### Keap1 70kDa





**Repeat1**





**Repeat2**





**Repeat3**

### Nucl-p65 65kDa

**

**

**Repeat1**





**Repeat2**





**Repeat3**

### cyto-p65 65kDa

**

**

**Repeat1**





**Repeat2**





**Repeat3**

### p-ikbα 36kDa


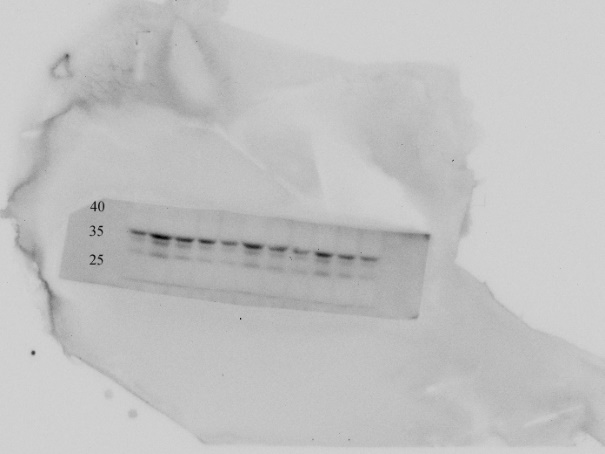


**Repeat1**





**Repeat2**





**Repeat3**
